# Barriers to oral healthcare for autistic children in China

**DOI:** 10.3389/fpsyt.2026.1910959

**Published:** 2026-08-26

**Authors:** Xing Wang, Xuchen Huang

**Affiliations:** 1 Department of Stomatology, China Rehabilitation Research Center, School of Rehabilitation, Capital Medical University, Beijing, China; 2 Department of Stomatology, Beijing Chao-Yang Hospital of Capital Medical University, Beijing, China

**Keywords:** autism, barriers, China, health inequity, oral healthcare

## Introduction

Access to oral healthcare is essential for general health and well-being. For autistic children, however, even routine dental attendance can be challenging. Sensory sensitivities, unfamiliar environments, and communication differences may make conventional dental visits particularly stressful (1). Yet the barriers to appropriate care are multifactorial. While the healthcare system’s failure to make sufficient adjustments for neurodevelopmental differences is a central factor, oral health outcomes for autistic children also depend on interactions among the child’s individual characteristics, family and social circumstances, and the dental care system’s ability to provide reasonable accommodations, procedural preparation, and person-centred care. In China, the shortage of dentists trained in special care dentistry, together with the concentration of specialized pediatric services in tertiary hospitals in major cities, means that many families struggle to find appropriate care.

China has made meaningful strides in disability policy in recent years, including the Healthy China 2030 framework (2) and the Action Plan for Caring for Autistic Children (3). However, current disability strategies remain largely focused on the disabilities themselves, while secondary health domains critically affected by disability receive less attention. This article examines the barriers autistic children encounter when seeking oral healthcare in China, while recognizing the progress that has been made, and considers how policy frameworks might be further adapted to address these inequities.

## Oral health needs and associated factors among autistic children

Autism spectrum disorder affects approximately 0.70% of children aged 6 to 12 years in China, according to the first national prevalence study based on multi-center population data (4). Oral health problems are common among autistic populations. Difficulties with tooth brushing due to sensory aversions, use of medications that cause dry mouth, and dietary preferences for soft or sweet foods may contribute to elevated oral health risks in autistic children. A Mendelian randomization study found that while pervasive developmental disorder showed a causal relationship with permanent tooth caries, autism spectrum disorder itself did not demonstrate a direct causal effect on either primary or permanent tooth caries. This suggests that atypical eating behaviors and dietary patterns, rather than ASD per se, may play a more significant role in the elevated caries risk observed in this population (5).

A review of oral health research on autistic children in China confirms that this group experiences poorer oral hygiene, higher detection rates of dental calculus, and more frequent cases of gingivitis compared with neurotypical peers (6). Another study of 117 children with autism and 121 neurotypical peers in China found that 74.36% of autistic children had dental caries and 52.14% had periodontal problems. Only 17.09% brushed their teeth twice or more per day, and 13.68% consistently used toothpaste. The study also found that 93.16% of autistic children were cared for full-time by a parent, and factors affecting oral health included brushing habits, primary caregivers, and parental education level (7). These findings suggest that the oral health challenges faced by autistic children are not merely clinical problems but have broader social and economic dimensions that affect entire families. Recognizing the magnitude of these needs is an important first step toward developing appropriate policy responses.

## Workforce preparedness and professional training

Many dentists receive limited training in the management of autistic patients during their undergraduate education. A review of oral health research on autistic children notes that multiple adverse factors, including sensory sensitivities, communication barriers, and a shortage of dentists trained in special care dentistry, collectively contribute to poorer oral health outcomes in this population (6). Consequently, newly qualified dentists may lack confidence in adapting their clinical approach for autistic children.

Behavioral management techniques that are effective for neurotypical children, such as tell show do, may require modification for autistic patients who have communication differences or difficulty processing verbal instructions. Sensory adaptations to the clinical environment, such as dimming lights or minimizing unexpected noises, are straightforward to implement but are not routinely taught or practised in many dental education programmes. Without such adaptations, dental visits may become negative experiences for autistic children, reinforcing avoidance behaviors and making future care increasingly difficult.

Continuing professional education in special care dentistry remains limited in China. In April 2025, the National Center for Mental Health and the Chinese Stomatological Disease Prevention Foundation jointly launched the Star Lighting Programme, a public welfare initiative aimed at improving oral healthcare for autistic children. The programme is structured as a pilot continuing professional education initiative. Its first phase covered 20 institutions across the country. The curriculum includes training for dental practitioners on communication strategies, environmental sensory adaptations, and behavioral management techniques, as well as oral health education for families and special education institutions (8). However, this initiative remains in its early stages, and detailed outcome data are not yet publicly available. Specific clinical guidance on environmental adaptations, communication strategies, and behavioural management techniques has not yet been developed into detailed, implementable protocols. The gap between the needs of this growing patient population and the preparedness of the dental workforce continues to represent a significant barrier to equitable care. The identified gap, the growing autistic population and insufficient professional preparation, requires a more differentiated understanding of training needs. Undergraduate dental curricula, continuing professional education, and postgraduate specialization represent distinct levels of professional preparation and should not be treated as equivalent.

Health authorities and academic institutions should define these levels of care. Basic competencies, including communication strategies, sensory accommodations, behavioral support, prevention, and routine dental management, should be incorporated into undergraduate curricula and continuing education for general dental practitioners. Advanced competencies for complex cases that warrant referral should be developed through postgraduate training in special care dentistry. Clear referral pathways should be established to guide general practitioners in identifying patients who require specialized care.

## Geographic and financial inequities

Specialized dental services for autistic children, particularly those offering general anaesthesia or sedation, are concentrated in major cities and tertiary hospitals. Families in rural or smaller urban centers may need to travel considerable distances for appropriate care. Even where services exist, waiting lists can be lengthy.

A documented case from a Beijing hospital illustrates these challenges. A ten-year-old autistic child with moderate autism and intellectual disability suffered traumatic tooth avulsion at school. The family visited multiple hospitals but were turned away due to the child’s inability to cooperate with routine procedures. An initial re-implantation procedure required four clinicians and the mother to physically restrain the child (9). Similar difficulties are reported elsewhere. A mother in Shenzhen, a major first-tier city, took her 30-year-old autistic daughter to multiple hospitals over several years, but all attempts failed due to the daughter’s inability to cooperate. The mother eventually resorted to learning dental skills online and filling the cavities herself (10). While these individual cases do not represent all experiences, they exemplify how fragmented care pathways can jeopardize even emergency dental treatment for autistic children, and that access remains a problem even in cities with advanced medical resources.

Financial barriers further compound access issues. Basic medical insurance coverage for dental treatment varies substantially across provinces. General anaesthesia for dental procedures, often necessary for autistic children who cannot tolerate chair side treatment, is frequently excluded or requires substantial out-of-pocket payment. For families already managing the costs of behavioral therapy and other supports, this financial burden may be prohibitive. National data confirm that dental care utilization among preschool children is concentrated in higher socioeconomic status families, with household income accounting for 45.66% of observed inequality (11). For autistic children, who may require more intensive and costly dental care, this socioeconomic gradient may be even steeper.

## Local initiatives and their limitations

On the positive side, some public dental hospitals in southern China have taken the lead in offering accessible dental services for autistic patients (12). Public welfare events such as free oral health education and fluoride application also represent progress (13). However, these are isolated examples. Most of these pioneering institutions are located in the wealthier southern region of the country (10). Moreover, free check-ups and fluoride application, while beneficial, are not the same as providing treatment for existing dental problems. The main challenge is therefore not the complete absence of services, but rather their limited coverage, uneven regional distribution, and insufficient integration into the basic medical insurance system.

For most families, relying on the basic medical insurance system to cover specialised dental care for their autistic children remains difficult. Private hospitals can offer good services, but families must pay fully out of pocket, and the costs are considerably higher than standard care (9). Few autistic children have families that can afford this option. Without policy changes at the national level, these issues are unlikely to be resolved. The number of families that can benefit from the current patchwork of local initiatives remains very limited.

## Policy frameworks and implementation gaps

China has made notable progress in disability policy in recent years. The Action Plan for Caring for Autistic Children (3) focuses on early intervention, education, and rehabilitation services, but makes no specific mention of oral health. The Star Lighting Programme represents progress, but it remains a voluntary public welfare effort rather than a mandatory component of the healthcare system (8). In its first phase, it covered only 20 institutions, far from sufficient to meet the needs of the autistic population across the country.

A further gap concerns financing. China has established rehabilitation subsidy systems for autistic children across different regions. For example, Shenzhen offers an annual subsidy at the higher end of the national range (14). These funds are primarily allocated for behavioral therapy and special education services. They are not designed to cover dental procedures. In principle, autistic children may access basic medical insurance for hospitalization, and some regions have included autism in the catalogue of outpatient special chronic diseases. However, dental treatment under general anaesthesia often involves navigating complex reimbursement pathways. Without clearer policy guidance to include dental general anaesthesia explicitly under basic insurance or to increase dedicated funding for this population, systemic improvements remain unlikely.

## Pathways towards more equitable care

Several policy and practice adjustments could begin to address these barriers. None requires radical system redesign, but each would benefit from sustained attention and coordination across different actors.

First, professional associations could develop and disseminate clear clinical guidance for the dental management of autistic children. Such guidance could cover environmental adaptations, communication strategies, and indications for referral for general anaesthesia. While Chinese researchers have summarized management strategies for dental anxiety and uncooperative behaviors in autistic children, a formal clinical guideline is still lacking. Professional societies could take the lead in developing one, drawing on international models such as the British Society of Paediatric Dentistry’s oral health advice leaflet for parents and carers of autistic children (15), while recognizing that formal clinical guidelines tailored for dental professionals are still needed in China. Adaptation to the Chinese context would require consideration of the country’s primary care-based dental service structure, regional disparities in specialist availability, and the existing framework of disability insurance. For example, referral pathways would need to be designed around China’s three-tier hospital system, with basic management protocols appropriate for community dental clinics and advanced care concentrated in specialist centers.

Second, undergraduate dental curricula could incorporate more substantial training in special care dentistry. Even modest increases in teaching hours might improve graduates’ competence. However, education alone may not be enough. A European study found that while prior education on special care dentistry increased dentists’ willingness to treat patients with disabilities, more than half of responding dentists had never received such training, and dentists were less willing than dental hygienists to engage in future initiatives. The authors concluded that educational efforts must be supported by governmental actions and regulations to be effective (16). This author has previously argued that the oral health needs of the wider disabled population have long been neglected in China, and that the issue extends beyond autism to the entire disabled population (17). Continuing education courses could be offered to practising dentists through provincial dental associations, but without policy support and regulatory incentives, uptake may remain low.

Third, the family doctor contracting system could be extended to include oral health assessments as a routine component for disabled children. This would involve updating service packages and training primary care providers in basic oral health screening and referral protocols. The rationale is that autistic children often struggle with unfamiliar environments and people. A familiar family doctor who knows the child’s sensory sensitivities and communication patterns may be better positioned to provide preventive oral care than a series of unfamiliar specialists. Continuity of care has been identified as important for this population (18).

Fourth, public financing mechanisms could be adjusted to reduce out-of-pocket costs for essential dental care for autistic children. This might involve expanding insurance coverage for general anaesthesia for dental treatment where clinically justified, or establishing regional specialized dental centers with adequate funding to serve catchment areas without imposing prohibitive costs on families.

Fifth, digital tools and visual pedagogy could be harnessed to improve oral health education for autistic children and their families. A randomized controlled trial in India found that a tooth brushing visual pedagogy using sequential photographs significantly improved brushing skills, oral hygiene, and gingival health in 200 autistic children aged 6 to 17 years (19). Video modelling and sensory-adapted environments have also been shown to reduce distress during dental visits for children with ASD. These approaches could be adapted for the Chinese context, for example through mobile applications or video-based training programmes delivered via social media platforms. Professional associations and public health authorities could promote such tools as part of a broader strategy to support families.

## Conclusion

Recent policy initiatives such as the Action Plan for Caring for Autistic Children and the Star Lighting Programme reflect growing official awareness of this issue. However, oral health remains underaddressed in current disability policies, with no explicit mention in the Action Plan and only voluntary pilot-level attention through the Star Lighting Programme. Regional services in some public dental hospitals offer promising models, but these are limited in number and concentrated in wealthier southern cities. Persistent challenges include insufficient training in special care dentistry, weak financial protection for dental general anaesthesia under basic medical insurance, and fragmented care pathways. Bridging this gap will require coordinated efforts to embed oral health into disability policies, expand training, and adjust financing mechanisms. Without such systemic changes, the current pattern of uneven, voluntary initiatives will not meet the scale of the need.
